# An assessment of existing models for individualized breast cancer risk estimation in a screening program in Spain

**DOI:** 10.1186/1471-2407-13-587

**Published:** 2013-12-10

**Authors:** Arantzazu Arrospide, Carles Forné, Montse Rué, Núria Torà, Javier Mar, Marisa Baré

**Affiliations:** 1 Gipuzkoa Health Research Unit. Alto Deba Integrated Health Organization, Mondragon, Spain; 2 Health Services Research Network in Chronic Diseases (REDISSEC), Spain; 3 Basic Medical Sciences Department, Biomedical Research Institute of Lleida, IRBLLEIDA-University of Lleida, Catalonia, Spain; 4 Epidemiology Unit, Breast cancer early detection program, UDIAT. Parc Taulí Sabadell-University Hospital, Parc Taulí s/n, 08208, Sabadell, Catalonia, Spain; 5 Clinical Management Service, Alto Deba Integrated Health Organization, Mondragon, Spain; 6 Pediatrics, Obstetrics and Gynecology and Preventive Medicine, Universitat Autònoma de Barcelona (UAB), Catalonia, Spain

**Keywords:** Breast cancer, Screening, Risk models, Individual risk, Breast density

## Abstract

**Background:**

The aim of this study was to evaluate the calibration and discriminatory power of three predictive models of breast cancer risk.

**Methods:**

We included 13,760 women who were first-time participants in the Sabadell-Cerdanyola Breast Cancer Screening Program, in Catalonia, Spain. Projections of risk were obtained at three and five years for invasive cancer using the Gail, Chen and Barlow models. Incidence and mortality data were obtained from the Catalan registries. The calibration and discrimination of the models were assessed using the Hosmer-Lemeshow C statistic, the area under the receiver operating characteristic curve (AUC) and the Harrell’s C statistic.

**Results:**

The Gail and Chen models showed good calibration while the Barlow model overestimated the number of cases: the ratio between estimated and observed values at 5 years ranged from 0.86 to 1.55 for the first two models and from 1.82 to 3.44 for the Barlow model. The 5-year projection for the Chen and Barlow models had the highest discrimination, with an AUC around 0.58. The Harrell’s C statistic showed very similar values in the 5-year projection for each of the models. Although they passed the calibration test, the Gail and Chen models overestimated the number of cases in some breast density categories.

**Conclusions:**

These models cannot be used as a measure of individual risk in early detection programs to customize screening strategies. The inclusion of longitudinal measures of breast density or other risk factors in joint models of survival and longitudinal data may be a step towards personalized early detection of BC.

## Background

It is estimated that, in the year 2015, 21,000 women in Spain will be diagnosed with breast cancer (BC), representing 25% of all cancers among women [1]. BC is the cancer that results in the greatest global mortality among women (268,000 deaths, 12.7% of all deaths) [1]. Given that the majority of known risk factors for BC are not modifiable, population-based primary prevention programs do not exist. As a consequence, early detection is a priority among public health programs, with the goal of improving disease prognosis and reducing mortality. Early detection or screening programs, together with the development of new adjuvant treatments, have contributed to the reduction in mortality associated with BC [2,3].

Currently, age and gender are the only criteria for defining the target population to be screened. Nevertheless, it has been reported that age at first birth, family history of BC, mammographic density or genetic factors are also associated with greater risk [4,5]. Having a reliable individual BC risk estimate based on known factors makes it possible to develop personalized screening programs and optimize the use of resources in a population.

Taking individual risk into account in screening strategies is new. In the USA, the National Cancer Institute started an initiative, the Cancer Intervention and Surveillance Modeling Network (CISNET), with the goal of evaluating the impact of screening and adjuvant treatments on BC incidence and mortality [6-8]. Recently, in a cost-effectiveness study, Schousboe et al. [9] have proposed different screening periodicities based on BC risk, measured as a function of breast density, family history of BC and previous breast biopsy.

The estimate of BC risk has been the subject of the publication of several articles in recent decades. The model created by Gail et al. in 1989 is, without doubt, the most widely known and used up to this point for the prediction of BC [10]. Furthermore, this model has been the reference for other models developed more recently. The modification of the Gail model, by including breast density and the weight of the woman as risk factors, led to the model developed by Chen, et al. [11]. The model described in 2006 by Barlow, et al. [12] takes into account, in addition to breast density, factors such as hormone replacement therapy, body mass index, race or ethnicity, which had not been previously incorporated into models as predictive variables. There are other models that either estimate the risk of carrying mutations in the BRCA1 or BRCA2 genes or use the information on BRCA1/2 status to improve the estimates of BC risk. Models like BRCAPRO [13] or the Tyrer-Cuzick [14] model are primarily based on family history of breast and ovarian cancer.

The generalized use of risk models requires that they be previously validated in different populations, given the possible differences in the distribution of risk factors and in the epidemiology of BC. Once external validity is verified, personalized screening strategies based on risk can be designed with the aim of improving the efficiency of screening programs. The principal objective of this study was to evaluate the calibration and discriminatory power of predictive models of BC risk, without genetic information, in a cohort of women in a Spanish early detection program.

## Methods

This is a validation study of the main models developed for estimating the risk of BC for women not at high risk. The selected models were identified from the published literature. We included 13,760 women that participated for the first time in the BC early-detection program in the Sabadell-Cerdanyola (EDBC-SC) area in Catalonia (Spain), between October 1995 and June 1998. The participants did not have a personal history of BC and were followed for vital status or possible diagnosis of BC until July of 2010 [15,16]. The EDBC-SC screening program offers biennial mammography for women aged 50 to 69. The data for this study were obtained through a questionnaire administered on the first visit, which included demographic variables, weight and height, personal gynecological history and family history of BC. Moreover, as a remarkable and unique characteristic among the Spanish BC screening programs, breast density was recorded on each mammographic test and rated according to the Breast Imaging Reporting and Data System (BI-RADS) [17]. Of the 13,760 women interviewed, we excluded seven without follow-up data, as well as 29 women who were diagnosed with BC and 15 who died within 6 months of baseline. We analyzed incident invasive cancers diagnosed at any time during follow-up, whether the diagnosis was made within the program or took place outside of it [16]. The final sample included 13,709 women, with 329 diagnosed with invasive BC.

### Description and changes on the selected models

The models selected for evaluation were developed by Gail [10], Chen [11] and Barlow [12]. The Gail and Chen models have an identical structure. They estimate the risk of developing BC over time using three components: 1) age-specific relative risks for selected risk factors, 2) incidence of BC in a baseline study population, and 3) competing risks of death.

The original Gail model included both ductal carcinoma in situ (DCIS) and invasive BC. A few years later the incidence rates were modified with the objective of using the model for invasive BC only [18,19]. Chen and Barlow considered only invasive BC in their respective models. Since the selected models, except the initial Gail model, were developed to predict the risk of invasive BC, in this study we have considered only invasive BC. We customized the Gail and Chen models using an estimated incidence function of invasive BC in Catalonia. Women diagnosed with DCIS in our study cohort were not excluded from the analysis, they were considered at risk of developing invasive BC.

To obtain the baseline BC risk of the study population, required for the Gail and Chen models, BC incidence was multiplied by the complement of the attributable risk (1-AR) corresponding to the distribution of risk factors in the study sample. The AR calculation was performed as described in Chen et al. [11]. We used the relative risks of the covariates that were estimated when the models were developed. Since the AR varied little with age, it was considered a constant value for the whole range of ages. The estimated AR for the Gail model was 0.369, and for the Chen model, 0.805. The difference in AR between the Gail and Chen models was due to the fact that the Chen model includes breast density and therefore the baseline risk is considerably lower.

Incidence data for invasive BC were obtained from the Girona and Tarragona Cancer Registries. Incidence rates for the observed period and projected rates for subsequent years were estimated using an age-cohort model with age as a fourth degree and cohort as quadratic polynomials (see Additional file 1: Table S1). Mortality rates in the study population were obtained from the Mortality Registry of the Catalan Department of Health (see Additional file 1: Table S1). The mortality rates from causes other than BC, by age and cohort, were obtained from Vilaprinyó et al. [20].

To estimate the relative risks of BC, the Gail model takes into account the number of first degree relatives with a history of BC, age at first live birth, age at menarche and the number of previous benign biopsies. The Chen model also includes breast density and weight, but unlike the initial Gail model does not include the age at menarche or interactions. The Barlow model includes breast density, hormone replacement therapy, body mass index, result of previous mammography exams, race and ethnicity as risk factors. For the Barlow model, the projected risk of BC was based on two separate logistic regression models, one for pre-menopausal women and the other for postmenopausal women.

Projections of risk were obtained at 3 and 5 years, starting six months after the first screening mammogram. Although most of the studies in the literature have worked with five years of follow-up, we considered that projection at 3-years would be useful for short-term decision-making on screening. For the Barlow model, which was designed to estimate the risk of developing invasive BC in a period of one year, the original article recommends projecting the risk for longer periods assuming that the probability of developing BC is identical and independent in each of the ensuing years [12]. Risk estimates for the three models were obtained using Mathematica [21].

### Statistical analysis

We performed a descriptive analysis of the studied variables. Characteristics of women in relation to BC diagnosis were compared using the chi-square test or the Fisher’s exact test for dichotomous variables.

The calibration of the models was assessed using the Hosmer-Lemeshow goodness-of-fit C statistic [22]. The C statistic compares the observed (O) and expected (E) number of BC cases by risk quantiles. The expected number of cases was obtained by adding the probabilities estimated by the models for each woman in the group. First, calibration was assessed by quintiles of risk, for the 3 and 5-year projections. Although deciles are often used, we considered that quintiles were more appropriate, given the small number of cancer cases. Then, for the 5-year projections, calibration was assessed on groups determined by categories of risk factors. Trends in the E/O ratio by categories of risk were assessed using the chi-square test for trends in order to search for subgroups in which the models worked the best.

The model’s discrimination was assessed using the Harrell C statistic, which measures the proportion of all patient pairs in which the predicted breast cancer probability and the follow-up interval (or time to event if the final event occurs), are ranked equally [23,24]. This concordance measure is a modification of the area under the receiver operating characteristic curve (ROC) that we also included in order to compare our results with similar studies. For these analyses we used the Stata/SE software [25].

## Results

Table 1 shows the main characteristics of the studied women. The mean age was 57.0 years and 94.4% of them were postmenopausal. The 18.6% of the women in the study had their first menstrual period before age 12 and the 46.6% of women had their first child at ages between 20 and 24 years. In the study sample, 7.9% of women who subsequently developed invasive BC had first-degree relatives with BC while this percentage was 5.3% in women who had not developed BC. This difference was not statistically significant. However, the differences in breast density, age at first mammogram and previous benign breast disease were significant. Many women reported having previous benign breast disease with no previous biopsy. This was not an unusual practice, in the past, in our publicly funded health system.

**Table 1:** Characteristics of the study sample

		**No cancer**^**(1) **^**(N = 13380)**		**Cancer (N = 329)**		**Total (N = 13709)**		
		**N**	**%**	**N**	**%**	**N**	**%**	**p-value**^**(3)**^
Age at first mammogram, y	50-54	4975	37.18	120	36.47	5095	37.17	0.026
	55-59	3602	26.92	110	33.43	3712	27.08	
	60-64	4216	31.51	83	25.23	4299	31.36	
	65-69	587	4.39	16	4.86	603	4.40	
Age at menarche, y	<12	2493	18.63	62	18.84	2555	18.64	0.990
	12-13	5243	39.19	129	39.21	5372	39.19	
	> = 14	5505	41.14	134	40.73	5639	41.13	
	Unknown	139	1.04	4	1.22	143	1.04	
Age at first live birth, y	<20	1065	7.96	26	7.90	1091	7.96	0.050
	20-24	6242	46.65	151	45.90	6393	46.63	
	25-29	4109	30.71	91	27.66	4200	30.64	
	>29	966	7.22	37	11.25	1003	7.32	
	No children	902	6.74	23	6.99	925	6.75	
	Unknown	96	0.72	1	0.30	97	0.71	
Body mass index	<25	3347	25.01	92	27.96	3439	25.09	0.569
	25-29	4519	33.77	105	31.91	4624	33.73	
	30-34	1944	14.53	53	16.11	1997	14.57	
	> = 35	675	5.04	16	4.86	691	5.04	
	Unknown	2895	21.64	63	19.15	2958	21.58	
Breast density (BI-RADS)^(2)^	1	2969	22.19	50	15.20	3019	22.03	<0.001
	2	5378	40.19	102	31.00	5480	39.98	
	3	2338	17.47	83	25.23	2421	17.65	
	4	2067	15.45	73	22.19	2140	15.63	
	Unknown	628	4.69	21	6.38	649	4.73	
No. of biopsies	0	13263	99.13	325	98.78	13588	99.12	0.513
	> = 1	117	0.87	4	1.22	121	0.88	
Menopausal status	No	747	5.58	18	5.47	765	5.58	0.930
	Yes	12633	94.42	311	94.53	12944	94.42	
Affected first-degree relatives	No	12647	94.52	302	91.79	12949	94.46	0.195
	Yes	704	5.26	26	7.90	730	5.32	
	Unknown	29	0.22	1	0.30	30	0.22	
Previous benign breast disease	No	12420	92.83	292	88.75	12712	92.73	0.005
	Yes	960	7.17	37	11.25	997	7.27	

^(1)^Ductal carcinoma in situ cases were included as no cancer cases.

^(2)^1: Almost entirely fat, 2: Scattered fibroglandular densities, 3: Heterogeneously dense, 4: Extremely dense.

^(3)^P-values have been obtained excluding the unknown values.

Median follow-up time was 13.3 years with an interquartile range of 12.7-13.9 years.

### Validation of the Gail, Chen and Barlow models

The Gail and Chen models showed good calibration, at 3- and 5-years, with similar expected and observed number of cases and p-values >0.05 for the Hosmer-Lemeshow C statistics (Table 2). Conversely, the Barlow model overestimated the number of cases, with ratios E/O above 1.8 in all the quintiles of risk and values above 3.3 in the upper quintiles.

**Table 2:** Calibration of the risk models by quintiles of risk

		**N**	**Expected cases (E)**	**Observed cases (O)**	**E/O**	**C Hosmer-Lemeshow statistic**	**p-value**
Gail model							
3-year	1	2569	10	9	1.11		
	2	2894	13	14	0.93		
	3	2656	13	12	1.08		
	4	2740	16	11	1.45		
	5	2587	20	17	1.18	2.28	0.516
5-year	1	2671	17	16	1.06		
	2	2705	21	22	0.95		
	3	2612	22	16	1.38		
	4	2742	27	18	1.50		
	5	2716	37	35	1.06	4.90	0.180
Chen model							
3-year	1	2524	7	11	0.64		
	2	2420	9	7	1.29		
	3	2513	12	13	0.92		
	4	2498	15	15	1.00		
	5	2479	22	14	1.57	5.76	0.124
5-year	1	2423	12	14	0.86		
	2	2519	17	11	1.55		
	3	2495	21	21	1.00		
	4	2511	26	23	1.13		
	5	2487	39	28	1.39	5.97	0.113
Barlow model							
3-year	1	2713	19	8	2.38		
	2	2722	29	13	2.23		
	3	2716	39	10	3.90		
	4	2804	50	13	3.85		
	5	2754	70	19	3.68	103.22	< 0.001
5-year	1	2713	31	17	1.82		
	2	2847	51	18	2.83		
	3	2591	62	18	3.44		
	4	2833	84	20	4.20		
	5	2725	115	35	3.29	168.49	< 0.001

When comparing the means of the estimated risk values by BC diagnosis, there were statistically significant differences in the three models at 5-years, but not at 3-years (Table 3).

**Table 3:** Means of the probabilities of developing breast cancer and discriminatory power of the models

		**Gail model**	**Chen model**	**Barlow model**
3-year	No cancer	0.005340	0.005320	0.015089
	Cancer	0.005728	0.005624	0.015514
	p-value	0.100	0.354	0.648
	AUC	0.562 (0.481, 0.644)	0.523 (0.441, 0.604)	0.526 (0.448, 0.603)
	C-Harrell	0.562 (0.481, 0.643)	0.523 (0.442, 0.603)	0.526 (0.449, 0.603)
5-year	No cancer	0.009226	0.009185	0.024974
	Cancer	0.010014	0.010723	0.028571
	p-value	0.011	< 0.001	0.002
	AUC	0.561 (0.499, 0.623)	0.586 (0.526, 0.646)	0.575 (0.513, 0.638)
	C-Harrell	0.561 (0.480, 0.642)	0.586 (0.526, 0.645)	0.575 (0.513, 0.637)

The studied risk models showed poor discrimination in the study sample. The areas under the receiver operating characteristic curve (AUC) ranged from 0.52 to 0.59. For the Gail’s model, the AUC confidence intervals for the 3- and 5-year projections included the value 0.50, which indicates the absence of discrimination. The Chen and Barlow models had higher discrimination at five years, with AUCs around 0.58, whereas the Gail model had an AUC around 0.56 in both the 3- and 5-year projections.

When time to BC diagnosis was taken into account, the Harrell C statistic indicated that the 5-year projection for the Gail model correctly ordered 56.1% of all pairs of women in the study. The 5-year projection for the Barlow and Chen models increased this figure to 57.5% and 58.6%, respectively (Table 3).

Table 4 shows the calibration by categories of the risk factors in the studied models. As before, the Gail and Chen models showed good calibration, except for age at first mammogram where the E/O ratio fluctuated. The Barlow model overestimated the number of BC cases and no trends were observed in the categories of the risk factors. By age groups, both the Gail and Chen models overestimated the number of cases in women 50–54 and 60–64 and underestimated them in women 65 years old or older. With regard to breast density, although the Gail and Chen models passed the calibration tests, the Gail model overestimated the number of cases in women with breast densities 1 and 2 and the Chen model in women with breast density 4.

**Table 4:** Calibration of the risk models by categories of the risk factors

				**Gail model**			**Chen model**			**Barlow model**		
		**N**	**Observed cases (O)**	**Expected cases (E)**	**E/O**	**p-value**^**(2)**^	**Expected cases**	**E/O**	**p-value**^**(2)**^	**Expected cases**	**E/O**	**p-value**^**(2)**^
Age at first mammogram, y	50-54	5095	31	42	1.35	0.009	41	1.32	0.006	99	3.19	< 0.001
	55-59	3712	34	33	0.97		31	0.91		97	2.85	
	60-64	4299	30	42	1.40		37	1.23		128	4.27	
	65-69	603	13	7	0.54		6	0.46		19	1.46	
Age at menarche, y	<12	2555	25	25	1.00	0.364	21	0.84	0.552	61	2.44	< 0.001
	12-13	5372	39	50	1.28		45	1.15		134	3.44	
	> = 14	5639	43	49	1.14		48	1.12		144	3.35	
Age at first live birth, y	<20	1091	9	7	0.78	0.181	7	0.78	0.370	24	2.67	< 0.001
	20-24	6393	45	53	1.18		50	1.11		151	3.36	
	25-29	4200	30	42	1.40		38	1.27		104	3.47	
	>29	1003	14	13	0.93		11	0.79		32	2.29	
	No children	925	10	9	0.90		9	0.90		30	3.00	
Body mass index	<25	3439	24	32	1.33	0.200	27	1.13	0.470	85	3.54	< 0.001
	25-29	4624	32	42	1.31		40	1.25		118	3.69	
	30-34	1997	19	18	0.95		19	1.00		53	2.79	
	> = 35	691	5	6	1.20		7	1.40		19	3.80	
Breast density (BI-RADS)^(1)^	1	3019	21	27	1.29	0.063	18	0.86	0.080	37	1.76	< 0.001
	2	5480	36	49	1.36		42	1.17		131	3.64	
	3	2421	29	22	0.76		26	0.90		81	2.79	
	4	2140	17	19	1.12		29	1.71		72	4.24	
No. of biopsies	0	13588	108	123	1.14	-	113	1.05	-	339	3.14	-
	> = 1	121	0	1	-		1	-		4	-	
Affected first-degree relatives	No	12949	97	111	1.14	0.149	105	1.08	0.605	317	3.27	< 0.001
	Yes	730	10	13	1.30		10	1.00		23	2.30	
Previous benign breast disease	No	12712	98	115	1.17	0.162	105	1.07	0.754	307	3.13	< 0.001
	Yes	997	10	9	0.90		9	0.90		36	3.60	

^(1)^1: Almost entirely fat, 2: Scattered fibroglandular densities, 3: Heterogeneously dense, 4: Extremely dense.

^(2)^The p-values shown in the table correspond to the Hosmer-Lemeshow C statistic. All the p-values for the chi-square test for trend were higher than 0.1.

## Discussion

The principal result of this study is that when adapting the incidence and mortality rates, the Gail and Chen models were well calibrated to estimate the risk of invasive BC in a population of Spanish women who participated in a screening program, whereas the Barlow model significantly overestimated this risk. All the three predictive models show a limited level of discrimination, despite the fact that they have been previously used in the US to classify women into high and low risk groups [18]. In general, good performance was seen in the Gail and Chen models when the subgroups of women are defined by categories of risk factors.

It is relevant to point out that the use of these models in our study reproduces the original results in terms of discrimination. In the original article, Chen et al. already compared the discriminatory value of the Gail model against a new model that included breast density. In that case, the AUC for the 5-year prediction was 0.596 for the Gail model and 0.643 for the Chen model [11]. In general, it is considered that a prediction tool should have an AUC greater than 0.7 [22]. With adaptation to the population incidence and mortality rates, we obtained an AUC of 0.561 for the Gail model and 0.586 for the Chen model, for the same 5-year period. Actually, the confidence intervals of the area under the curve in our study contained the values of the original models. The original Barlow publication only showed the discriminatory value of the one-year predictive model, 0.624 [12]. In our study, this figure was 0.602 and the 95% CI (0.440, 0.765) also included the original AUC value.

At the European level, there are adaptations of the Gail model in concrete populations such as an Italian and a Spanish study [26,27]. One important aspect of these studies is that they include relative risks of the risk factors adapted to their study population. Furthermore, they also modify the incidence of BC as well as mortality by other causes. The risk factors included and the methodology applied for the projection of risks at five years was exactly the same as that used in the original Gail model. Discrimination levels of the Italian and the Spanish adapted models were 0.590 and 0.544, respectively. In the Italian study, the AUC was similar to the 0.586 that Gail found in his study population, whereas in the Spanish study, the AUC was lower and similar to our estimate. Another article published in the US [28] showed that the use of relative risks specific to Hispanic and non-Hispanic populations slightly improved discrimination. In our study, the relative risks were not estimated using the study population due to small frequencies in some of the groups defined by risk factors. Although the original relative risks seem to work well for the Gail and Chen models, they may explain in part the lack of calibration of the Barlow model.

Other facts that can explain why the Barlow model did not perform well are differences in the population characteristics, inclusion criteria, and timing projections. In contrast to our study sample, women included in the Barlow study were racially and ethnically diverse. The Barlow study sample included the incident cases detected by the first mammogram and was developed as a short-time prediction model. Additionally, the model does not use BC incidence rate or mortality by other causes. All these facts also may explain why the Barlow model overestimates risk of breast cancer in our population. A new model for assessing 5-year risk was developed later by the Breast Cancer Surveillance Consortium [29], which would be interesting to assess in a Spanish population in future studies.

In Darabi et al. [30], where the Gail model was evaluated using data from a Swiss study, the result was an AUC and 95% confidence interval of 0.548 (0.527, 0.568). Furthermore, they determined the improvement in prediction due to the incorporation of breast density and body mass index. The expanded model increased the AUC to 0.571 (0.545, 0.597). Our results show that the Chen and Barlow models, that also incorporate breast density, have slightly greater discriminatory power for prediction at five years than the Gail model.

We have identified three published studies in which one of the studied models, the Gail model, was applied to the Spanish population. Pastor-Climente et al. [31] estimated the risk of developing BC in a 5-year period, using the Gail model calculator available on the web, without adapting either incidence or mortality for other causes [32]. The sample used included only women that had been diagnosed with BC. The study concluded that only 42% of women diagnosed with BC had a high risk, defined as 1.67% or greater [18]. Thus, the original Gail model showed low sensitivity, and sensitivity is a required characteristic for a model to be used for decision-making in a screening context. Buron et al. [33], in a screening program context, assessed the utility of the original Gail model to predict BC in women with a prior positive mammogram. At five years, discrimination was low (AUC = 0.61) and, using the standard threshold of 1.67%, sensitivity and specificity were 46.2% and 72.1%, also too low for clinical decision-making. The third study, by Pastor Barriuso et al. [27], assessed the performance of the original and a recalibrated Gail model together with a new model fully developed by the authors. Consistent with our results, the recalibrated Gail model was well calibrated overall, although it tended to underestimate risk for women in low-risk quintiles and to overestimate it in high-risk quintiles. In our study, we observed concordance between expected and observed in the low-risk groups and a slight overestimation of risk in high-risk quintiles.

Breast density is a risk factor strongly associated with the risk of BC, as demonstrated in recent years in various studies [34,35]. The Chen model was designed as an adaptation of the Gail model with the incorporation of breast density as a risk factor. If we compare the results obtained in our study, we see that the Chen model shows improved discrimination at five years over the Gail model, although in our sample the Chen model overestimates risk for women with high density. The Chen model used a quantitative measure of density, although it was then categorized into a variable with five categories, similar to the BI-RADS classification. Given the significant correlation between the BI-RADS and other quantitative measurement systems [36,37], and the availability of the BI-RADS in our screening program, we considered using it as an approximation. Nevertheless, the inclusion of longitudinal measurements of breast density in the models could improve the risk estimates, as other authors have shown [38].

Another risk factor with important weight in these models is family history. The coefficient of the Barlow model, for pre-menopausal women, is similar to the Chen model’s coefficient for the variable “number of first-degree relatives with BC”. Nevertheless, the Barlow model for post-menopausal women has a lower coefficient. It is possible that part of the risk attributable to family history is explained by other variables, such as body mass index or surgical menopause, which are not included in the other models mentioned. The Gail model, on the other hand, gives a higher weight to family history in comparison to the Chen model. With the inclusion of breast density in the model, family history loses its impact in risk prediction.

One of the principal contributions of our study is the assessment of the risk models using specific incidence and mortality rates by birth cohort in our geographic area. This procedure makes it possible to improve the Gail and Chen estimates based on the incidence rates of BC and mortality rates by other causes, which were obtained from a cross-sectional study. Given that BC incidence rates have an increasing trend, cross-sectional rates overestimate rates for past periods and underestimate those of future periods. As a result of using mortality rates by birth cohort, estimated survival in women over 50 in our study increased considerably in comparison with the US data of the original models. Therefore, a conclusion of our study was that, when local data for BC incidence and mortality from other causes were used, the Gail and Chen models provided unbiased estimates of risk of developing BC in our population.

One limitation of this study is that the Girona and Tarragona Cancer Registries do not include the population in the area studied. Although there were no differences observed in incidence rates between Girona and Tarragona, two areas of Catalonia that are geographically separated, it may be that the study area had a lower incidence of BC. Nevertheless, in a previous study, no differences were observed in BC mortality between a geographical region that included the study population, and the provinces of Girona and Tarragona [39].

Other limitations are related to the number of cancer cases and to missing values. As mentioned above, the small number of cancer cases precluded estimating specific relative risks, which have an impact on the performance of the models, along with the incidence and mortality rates. With respect to missing values, a sensitivity analysis with complete data showed that the calibration results were similar and discrimination slightly improved.

Finally, it is worth mentioning that the risk estimates are based only on the baseline characteristics reported at the first screening exam of the early detection program. With the number of previous biopsies being an important risk factor, a very small number of women reported having had biopsies before their first screening mammography. In these risk models, this is an important issue, because the estimating equation assumes that the probability or the relative risk is maintained over time.

## Conclusion

In conclusion, this work showed that using local data on BC incidence and mortality from other causes, appropriate group risk estimates for the Gail and Chen models can be obtained. Nevertheless, the three studied risk models do not have discriminatory power in our setting and therefore, they cannot be used as a measure of individual risk in early detection programs to customize screening strategies. More work is necessary in this field for obtaining reliable tools to estimate individual risk. The inclusion of longitudinal measures of breast density or other risk factors in joint models of survival and longitudinal data may be a step towards personalized early detection of BC.

## Competing interest

The authors declare that they have no competing interest in the research.

## Authors’ contributions

MB and MR conceived and coordinated the study, discussed the results and reviewed the manuscript. AA and CF performed the statistical analysis and drafted the manuscript. JM and NT discussed the results and reviewed the manuscript. All authors read and approved the final manuscript.

## Pre-publication history

The pre-publication history for this paper can be accessed here:

http://www.biomedcentral.com/1471-2407/13/587/prepub

## Supplementary Material

Additional file 1: Table S1 Incidence rates of breast cancer and mortality rates from other causes in Catalonia. Click here for file
