# Supplementary material for: An assessment of existing models for individualized breast cancer risk estimation in a screening program in Spain
Source: BMC Cancer. 2013 Dec 10;13:587. doi: 10.1186/1471-2407-13-587 (PMC4029404; doi:10.1186/1471-2407-13-587)
Supplement: Additional file 1: Table S1 — Incidence rates of breast cancer and mortality rates from other causes in Catalonia. [file 1471-2407-13-587-S1.docx]

**Supplementary table S1: Incidence rates of breast cancer and mortality rates from other causes in Catalonia.**

|  |  | Incidence rates per 100,000 women | | |
| --- | --- | --- | --- | --- |
| Birth cohort | | Year 1930 | Year 1940 | Year 1950 |
| Age | 45-49 | 93,7 | 120,6 | 145,6 |
|  | 50-54 | 121,8 | 156,8 | 189,3 |
|  | 55-59 | 150,5 | 193,8 | 234,0 |
|  | 60-64 | 184,3 | 237,4 | 286,5 |
|  | 65-69 | 227,4 | 292,8 | 353,4 |
|  | 70-74 | 280,3 | 360,9 | 435,6 |
|  | 75-79 | 334,5 | 430,7 | 519,9 |
|  |  | Mortality rates from other causes per 100,000 women | | |
| Birth cohort | | Year 1930 | Year 1940 | Year 1950 |
| Age | 45-49 | 229.5 | 157.7 | 105.1 |
|  | 50-54 | 286.8 | 196.9 | 131.1 |
|  | 55-59 | 385.4 | 266.4 | 178.6 |
|  | 60-64 | 550.5 | 383.8 | 259.8 |
|  | 65-69 | 828.0 | 532.2 | 397.6 |
|  | 70-74 | 1307.2 | 925.1 | 636.2 |
|  | 75-79 | 2164.4 | 1539.1 | 1063.6 |
